# Serum Peptidomic Profile as a Novel Biomarker for Rheumatoid Arthritis

**DOI:** 10.1155/2020/6069484

**Published:** 2020-08-03

**Authors:** Abeer A. Abdelati, Rehab A. Elnemr, Noha S. Kandil, Fatma I. Dwedar, Rasha A. Ghazala

**Affiliations:** ^1^Department of Internal Medicine, Rheumatology and Clinical Immunology Unit, Faculty of Medicine, Alexandria University, Alexandria 21612, Egypt; ^2^Department of Physical Medicine Rheumatology and Rehabilitation, Faculty of Medicine, Alexandria University, Alexandria 22511, Egypt; ^3^Department of Chemical Pathology, Medical Research Institute, Alexandria University, Alexandria 21523, Egypt; ^4^Department of Medical Biochemistry, Faculty of Medicine, Alexandria University, Alexandria 21648, Egypt

## Abstract

Over the last decades, there has been an increasing need to discover new diagnostic RA biomarkers, other than the current serologic biomarkers, which can assist early diagnosis and response to treatment. The purpose of this study was to analyze the serum peptidomic profile in patients with rheumatoid arthritis (RA) by using matrix-assisted laser desorption/ionization time-of-flight mass spectrometry (MALDI-TOF-MS). The study included 35 patients with rheumatoid arthritis (RA), 35 patients with primary osteoarthritis (OA) as the disease control (DC), and 35 healthy controls (HC). All participants were subjected to serum peptidomic profile analysis using magnetic bead (MB) separation (MALDI-TOF-MS). The trial showed 113 peaks that discriminated RA from OA and 101 peaks that discriminated RA from HC. Moreover, 95 peaks were identified and discriminated OA from HC; 38 were significant (*p* < 0.05) and 57 nonsignificant. The genetic algorithm (GA) model showed the best sensitivity and specificity in the three trials (RA versus HC, OA versus HC, and RA versus OA). The present data suggested that the peptidomic pattern is of value for differentiating individuals with RA from OA and healthy controls. We concluded that MALDI-TOF-MS combined with MB is an effective technique to identify novel serum protein biomarkers related to RA.

## 1. Introduction

Rheumatoid arthritis (RA) is the most common form of inflammatory arthritis that results in the destruction of articular cartilage and bone erosion [1]. Immunological studies revealed the presence of specific serological markers such as anti-cyclic citrullinated peptide antibody (ACPA) and rheumatoid factor (RF) that aid in the diagnosis of RA [2]. Indeed, the diagnosis is usually easy in established stages of the disease when the lesions are clinically and radiologically apparent. However, in early phases, when the introduction of appropriate therapy would be most useful, the diagnosis is usually challenging especially in early seronegative RA patients [3]. The 2010 American College of Rheumatology/European League Against Rheumatism (ACR/EULAR) classification criteria [4] for RA have been focused on features at earlier stages of disease rather than defining the disease by its late-stage manifestations. This will reinforce attention on the urgent need for earlier diagnosis to prevent, or at least decrease, the occurrence of the undesirable sequelae of RA.

Therefore, there has been an increasing need to discover new diagnostic biomarkers that can help early diagnosis and assist in evaluating disease activity, severity, and treatment response [5]. Over the last decade, the identification and quantification of novel biomarkers are a new area of interest in the clinical management of RA. Proteomics is the method of studying protein expression, structure, function, modifications, and interactions, as well as how these proteins change in different environments and conditions [6]. The discovery of proteomic technologies has led to advances in the analysis of synovial fluid, blood, and urine samples collected from RA patients. Furthermore, proteomics may help to identify newer autoantibodies and novel inflammatory acute-phase proteins beyond C-reactive protein (CRP) and serum amyloid A [7].

Different techniques have been used in proteomics. Among these techniques, matrix-assisted laser desorption/ionization time-of-flight mass spectrometry (MALDI-TOF MS) is emerging as a promising technology for the detection of complex proteins, as many protein mixtures of certain disorders have been identified [8]. Owing to its broader image, it allowed a better understanding of the intracellular protein composition, structure, and activity, thus aiding in the discovery of new biomarkers and achieving better comprehension of disease pathogenesis. Different types of biological samples collected from RA patients could be used including synovial tissue/fluid, blood, and urine [9]. Despite a wide range of availability of different protein species, serum represents a rich medium for the discovery of disease-specific biomarkers and helps identify new therapeutic targets.

In this context, several attempts have been made and some interesting proteins have been identified [10–12]. Yet, the integration of potential biomarkers resulting from proteomic analysis in RA is not fully established, and so, several studies are needed to confirm the efficacy of these approaches. The purpose of this study was to use MALDI-TOF-MS to identify differentially expressed disease-related peptide in patients' serum with RA.

## 2. Materials and Methods

This study was approved by the local ethics committee of the institution (no. 20/181), and written informed consent was obtained from each subject before the study. A total of 105 serum samples were included in this study, among which, there were 35 from patients with RA, 35 from patients with primary (idiopathic) OA as the disease control (DC), and 35 from healthy volunteers as the healthy control (HC) with matched age and sex. All RA patients were diagnosed according to the 2010 ACR/EULAR classification criteria for RA [4] and selected to be biologic disease-modifying antirheumatic drugs (bDMARDs) naïve. Osteoarthritis patients were selected with primary (idiopathic) OA of knee joints without any underlying cause of secondary OA and were classified by a five-grade scale according to the Kellgren and Lawrence (KL) radiographic classification scheme [13].

All patients were subjected to history taking, musculoskeletal examination, laboratory workup, as well as plain radiographic tests for hand and knee joints. In addition to the test of rheumatoid factor (RF) and anti-cyclic citrullinated peptide antibody (ACPA), calculation of the disease activity was performed for the RA group by utilization of the disease activity score-28 (ESR version) (DAS-28) [14].

All participants were subjected to serum proteomic profile analysis by using magnetic bead (MB) separation and MALDI [15].

### 2.1. Chemicals, Standards, and Consumables

Protein Calibration Standard I, Peptide Calibration Standard II (PepMix II), magnetic bead hydrophobic-interaction chromatography (MB-HIC C8) beads, and *α*-cyano-4-hydroxycinnamic acid were purchased from *Bruker Daltonics* (Bremen, Germany). Gradient-grade acetonitrile, ethanol, proanalysis-grade trifluoroacetic acid, urea, sodium chloride, and acetone were purchased from Sigma-Aldrich (St. Louis, Missouri, USA).

### 2.2. Serum Sample Collection, Storage, and Preparation

Peripheral venous blood samples were obtained from the participants in the morning and were drawn into tubes that were placed in an ice pack for transport. Each sample was centrifuged in a cooling centrifuge at 5°C for 15 minutes at 1800 × g. Then, serum was separated after acquisition by centrifugation and was aliquoted and stored immediately at -80°C until further analysis.

### 2.3. Peptidome Separation

Magnetic bead hydrophobic-interaction chromatography (MB-HIC C8) beads separate low molecular weight peptides according to the hydrophobicities (1-10 kDa). MB purifications were performed according to the manufacturer's protocol for serum. The peptide fraction was eluted from MB-HIC C8 beads with 5 *μ*l acetonitrile/water (1 : 1) from *Bruker Daltonics.*

### 2.4. MALDI-TOF-MS Analysis

MALDI-matrix *α*-cyano-4-hydroxycinnamic acid (CHCA) was chosen for the peptide profiling experiment on polished steel targets. One *μ*l of the sample was applied to a target spot and left to dry at room temperature; then, 1 *μ*l of the matrix was applied on the spot. The matrix consisted of CHCA (3 mg/ml) in 50% acetonitrile/2% TFA and prepared by mixing 1.2 mg HCCA, 200 *μ*l acetonitrile, 160 *μ*l deionized water, and 40 *μ*l of the 10% TFA.

The mixture was then left to dry at room temperature. Spectrum acquisition was done using the positive linear mode (1-10) kDa of the MALDI-TOF/TOF UltrafleXtreme mass spectrometer from Bruker Daltonics (Bremen, Germany). For optimum performance, the ClinPro standard (CPS) was used as a standard sample. Using the FlexControl™ software, peaks with a signal/noise (*S*/*N*) ratio above 3 were only chosen from the spectra generated.

### 2.5. Expression Profile Analysis and Statistical Analysis

The ClinPro Tools software 3.0 (Bruker, Daltonik, Germany) was used for processing and data analysis. The mean spectrum obtained from each subject data set was used for the statistical analysis. A difference with *p* value < 0.05 was considered statistically significant. A class prediction model was adjusted by applying 3 different machine-programming algorithms: Supervised Neural Network (SNN), genetic algorithm (GA), and Quick Classifier (QC) algorithms. Cross-validation was implemented to determine the accuracy of the class prediction [16].

## 3. Results

A total of 105 serum samples (35 RA patients, 35 OA patients, and 35 HC) were analyzed by MALDI. The detailed characteristics of the participants are shown in Table 1, and the disease parameters of the RA group are shown in Table 2.

### 3.1. MALDI-TOF-MS Spectrum Analysis and Model Generation

By using the spectral data from the three groups, three different classification models for the three groups were generated using GA, SNN, and QC algorithms. The GA model showed the best sensitivity and specificity in the three trials (RA versus OA, RA versus HC, and OA versus HC).

### 3.2. RA versus Disease Control (OA)

Among the peaks ranging from 1 to 10 kDa, 113 protein peaks significantly varied between RA and disease control (OA group) (*p* < 0.05) and discriminated sera of patients with RA from those with OA. Of which, 73 peaks were significant (*p* > 0.05) and 40 peaks were nonsignificant. Compared with the disease control group, 57 peaks were upregulated and 53 peaks were downregulated in the RA group, whereas 3 peaks were equally expressed in both groups. All these 113 protein peaks were entered into the ClinPro Tools software 3.0 to generate an optimal decision classification tree in the testing set. The machine learning genetic algorithm (GA) showed the most representative classification tree that comprised five integrated peaks with mass to charge ratios (*m*/*z*) of 75 : 7767.82, 40 : 2953.29, 41 : 2991.59, 48 : 4054.75, and 68 : 6434.51; all of them were significant and were selected as the best biomarkers of RA in the classification tree (Table 3).

The integrated peaks showed that all peaks were downregulated except peak 40 which was upregulated. External validation was performed for the three trials. The external validation of RA versus OA showed 97.8% sensitivity and 97.9% specificity.

**Figure 1:**
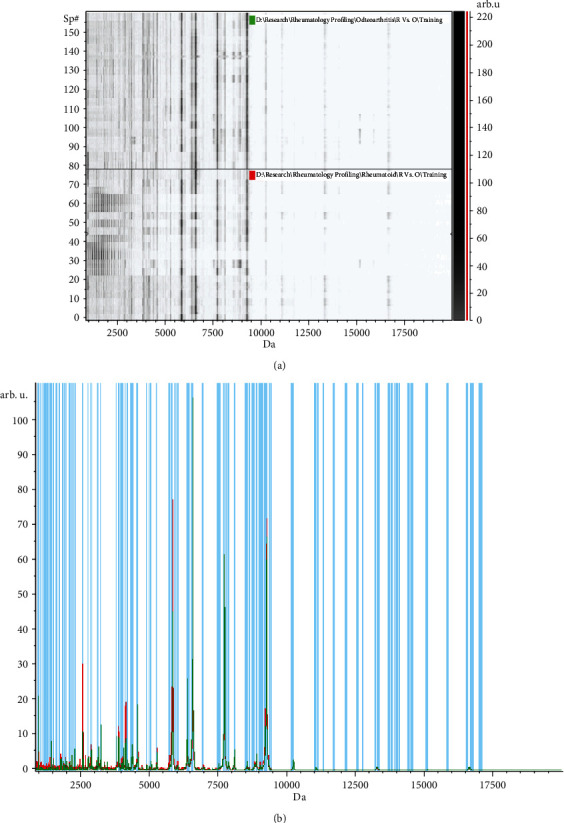
Peptidomic analysis of rheumatoid arthritis (RA) versus osteoarthritis (OA) group. (a) The pseudogel view using ClinPro Tools. The RA group at the bottom and OA group at the top. Each peak is represented by a vertical line. The difference in intensity between the lines represents the differential peak expression between the two groups. (b) The whole spectral view in ClinPro Tools. The figure represents the RA group in red against the OA group in green. Peaks included are demarcated with vertical blue lines. Peaks demarcated with red vertical lines are the integration regions.

The pseudogel view in ClinPro Tools was applied, and as shown in (Figure 1), each peak is represented by a vertical line. The difference in intensity between the lines in the two involved groups in the comparison represents the differential peak expression between every two groups of the three trials for RA versus OA, RA versus HC, and OA versus HC, respectively.

### 3.3. RA versus Healthy Control

**Figure 2:**
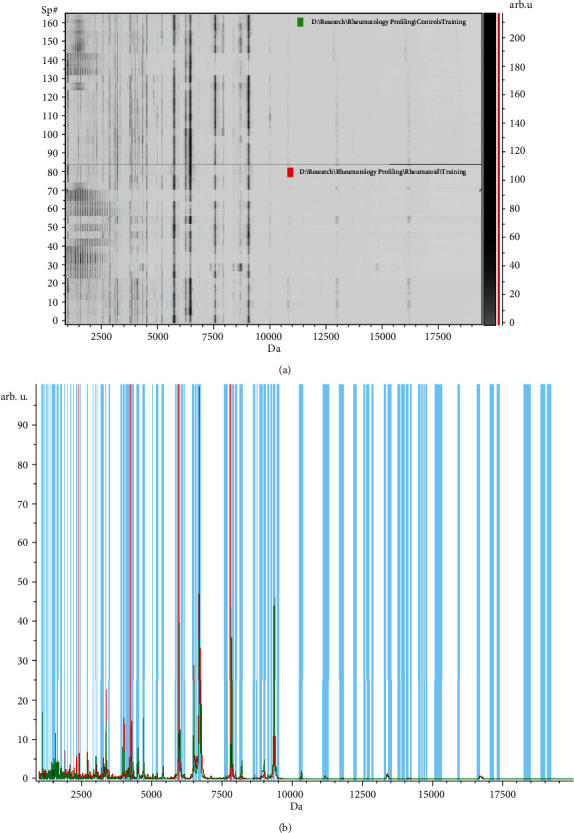
Peptidomic analysis of rheumatoid arthritis (RA) versus healthy control (HC) group. (a) The pseudogel view using ClinPro Tools. The RA group at the bottom and HC at the top. Each peak is represented by a vertical line. The difference in intensity between the lines represents the differential peak expression between the two groups. (b) The whole spectral view in ClinPro Tools. The figure represents the RA group in red against HC in green. Peaks included are demarcated with vertical blue lines. Peaks demarcated with red vertical lines are the integration regions.

The results revealed 101 peaks that discriminated RA from the control group; 53 were significant (*p* > 0.05), and 48 were nonsignificant. Sixty-two peaks were upregulated, and 39 peaks were downregulated. The GA model showed the most representative classification tree that comprised 5 integrated peaks that can discriminate between the two groups and might be the possible biomarkers in distinguishing RA from healthy subjects. Five peaks were integrated (with *m*/*z* of 25 : 2367.5, 62 : 7767.8, 53 : 5906.33, 14 : 1617.46, and 43 : 4210.99); 3 peaks (nos. 14, 25, and 62) were significant, and 2 were nonsignificant (nos. 43 and 53) (Table 3). All the integrated peaks were overexpressed except peak 62 which was downregulated (Figure 2). The sensitivity and specificity in the data of the training set were 97.5% and 95.3%, respectively.

### 3.4. OA versus Healthy Control

**Figure 3:**
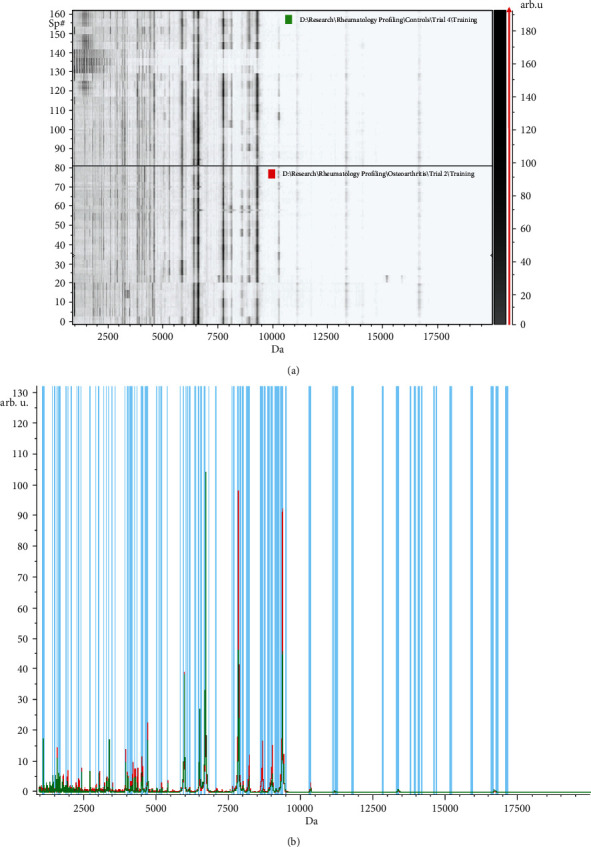
Peptidomic analysis of osteoarthritis (OA) versus healthy control (HC) group. (a) The pseudogel view using ClinPro Tools. The OA group at the bottom and HC at the top. Each peak is represented by a vertical line. The difference in intensity between the lines represents the differential peak expression between the two groups. (b) The whole spectral view in ClinPro Tools. The figure represents the OA group in red against HC in green. Peaks included are demarcated with vertical blue lines. Peaks demarcated with red vertical lines are the integration regions.

A total of 95 protein peaks were detected by MALDI-TOF-MS in the training set and identified the OA patients from the control group; 38 were significant (*p* > 0.05) and 57 nonsignificant. Fifty-seven peaks were upregulated, 33 were downregulated, and 5 were equally expressed in both groups. The GA model revealed five integrated peaks with *m*/*z* of 18 : 2953.08, 3 : 1077.37, 75 : 9289.67, 14 : 2272.81, and 23 : 3316.27; 3 peaks (18, 3, and 14) were significant, and peaks 75 and 23 were nonsignificant (Table 3). All the integrated peaks were upregulated except peaks 18 and 23 which were downregulated (Figure 3). The sensitivity and specificity in the data of the training set were 96.6% and 99.7%, respectively.

**Figure 4:**
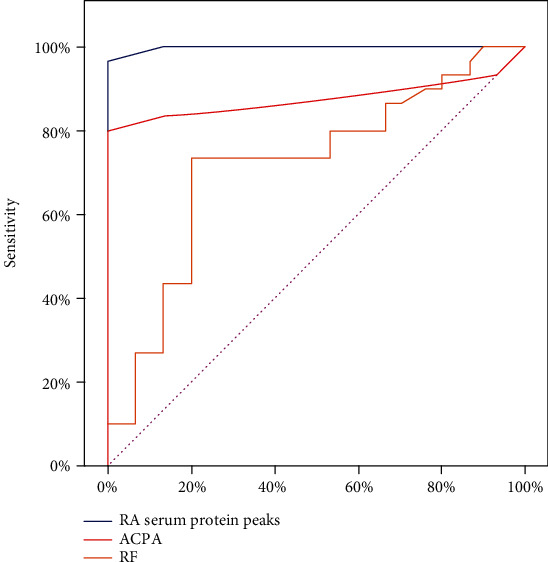
Receiver-operating characteristic (ROC) analysis of the training set samples comparing the diagnostic performance of rheumatoid arthritis (RA) serum protein peaks (AUC = 0.988), anti-cyclic citrullinated peptide antibodies (ACPA) (AUC = 0.875), and rheumatoid factor (RF) (AUC = 0.720) in diagnosing RA.

Results of the validation test for the training set data showed that the detected protein peaks could differentiate RA samples from those of the disease control and healthy control with sensitivity and specificity of 96.66% and 100.0%, respectively. It yielded the highest diagnostic value with an area under the curve (AUC) of 0.988 compared to other laboratory RA tests such as ACPA (AUC = 0.875) and RF (AUC = 0.720) (Table 4, Figure 4).

## 4. Discussion

In the management of RA, the interruption of the inflammatory cascade before it is fully established is the most effective. Therefore, it is evident that therapeutic intervention will have a greater effect on the outcome if started early and ideally if commenced before the occurrence of articular damage [17]. Therefore, the availability of new biomarkers will be useful in the diagnosis of early preradiographic disease and possibly the most promising way to improve RA management [18, 19].

Analysis of proteomic/peptidomic profile is one of the most promising methods for the identification of proteins and peptides connected with rheumatic diseases [20]. However, MALDI-TOF-MS cannot enable complete quantification of proteins. In this context, identifying the protein species depicted by the peaks on the spectra would provide further evidence that they are indeed biologically significant disease-related molecules [21].

In the current study, MALDI-TOF-MS combined with MB-HIC C8 were applied for identifying serum protein profiles, to establish a serological classification tree model, for RA patients in comparison with OA and HC. The low molecular weight proteins have been isolated by magnetic beads, and several considerable up- and downregulated proteins were recognized in the RA group compared to OA and HC groups. In our study, 113 peaks discriminated the RA group from the OA group with 5 integrated peaks (GA model); all integrated peaks were significant.

On performing the external validation for the three trials, RA-related protein peaks yielded a sensitivity of 97.8% and specificity of 97.9% when compared with the OA group and a sensitivity of 97.5% and specificity of 95.3% when compared with the HC group. On the other hand, the external validation of the OA group against the HC group revealed 96.6% sensitivity and 99.7% specificity. These findings support the ability of MALDI-TOF-MS coupled with magnetic beads to discover differentially expressed serum protein biomarkers in RA and OA patients, and this may reflect the differences in the pathogenesis between RA and OA.

In a previous study [22], differential proteomic and peptidomic analysis of plasma and synovial fluid was performed for patients with RA, OA, and reactive arthritis by applying mass spectrometric structure characterization of gel-separated proteins. Fibrin degradation products were detected in both groups. On the other hand, Calgranulin B (MRP14) and serum amyloid A have been exclusively identified in synovial fluid samples derived from RA and have not been observed in synovial fluids or plasma from OA patients.

In another study [23], exosomes were isolated from serum samples obtained from 43 subjects: 12 with active RA, 11 with inactive RA, 10 with OA, and 10 healthy donors. Two hundred and four (204) protein spots were detected by 2D-DIGE; among them, the protein spot identified as Toll-like receptor 3 (TLR3) showed approximately 6-fold higher intensity in the active RA group than in other groups. This may reflect the pathophysiology of active RA.

In the current study, in a comparison between RA and HC, 101 peaks were identified to be related to RA; among them, five peaks were integrated. This classification model could differentiate patients with RA from HC with a sensitivity of 97.5% and specificity of 95.3%. The peak patterns were almost similar among the samples for respective subjects in the repeated trials, indicating good reproducibility of the analysis.

In a similar study [24] involving the same range of protein detection 1-10 kDa, proteins were recognized by using MALDI-TOF-MS combined with WCX-MB-HIC C8, with a discrepancy between RA patients and the control group. The decision tree model included four protein peaks at *m*/*z* 4966.89, 5065.3, 5636.97, and 7766.87. Among the four peaks, *m*/*z* 4966.89 was overexpressed in RA cases, which might help in the diagnosis of RA by the detection of such protein peaks. On the contrary, three other protein peaks were downregulated. It is worth mentioning that a common peak of similar mass, 7767.8/7766.8, was observed to be integrated and downregulated in both studies in accordance with our work.

Additionally, Zhang et al. [25] studied proteomes in serum samples from 60 RA patients and 36 healthy controls using MALDI-TOF-MS, and a total of 33 peaks were identified to be related to RA, of which 5 peaks were used to be significant for RA diagnosis by pattern recognition software. The blind testing data indicated a sensitivity of 86.7% and a specificity of 90.0% for diagnosing RA. They reported that the decision model tree, based on the five candidate biomarkers, could provide a powerful and reliable diagnostic method for RA with high sensitivity and specificity.

An earlier study using the same technical approach identified protein biomarkers of early RA patients. Four peaks with *m*/*z* of 8133.85, 5844.60, 13541.3, and 14029.0 were recognized; the first three peaks were upregulated, while the last one was downregulated in RA compared to the control [26]. In another study, three peaks with *m*/*z* of 2490, 5910.07, and 6436.73 were identified in patients with RA, of which the first one was overexpressed and the last 2 were underexpressed in RA compared to controls. Moreover, the peaks with *m*/*z* of 1014.92 and 1061.38 were significantly overexpressed in the early RA group compared to those with established RA. This might provide an additional advantage of this technique that helps in differentiating early from established RA [27].

Yan et al. [8] used the same technique in RA and identified proteins with *m*/*z* of 3939, 5906, 8146, and 8569. These four proteins were again assessed for diagnostic accuracies and demonstrated a sensitivity of 100.0% and a specificity of 96.0% for differentiating RA patients from healthy controls. Their results had shown high levels of sensitivity and specificity, 100.0% and 81.2%, respectively. This finding is in line with our results, as peak number 53, with *m*/*z* 5906.3, was detected in our RA group and was reported by Yan et al. as well.

In the present study, 95 peaks were identified when comparing OA with HC. These peaks discriminated OA patients from the control group with 5 integrated peaks by the GA model. All were upregulated except peaks 18 and 23 which were downregulated. We thought that these differentially expressed protein peaks came from the production of fragments of extracellular matrix proteins as a result of structural damage in subchondral bone and articular cartilage degradation in OA. These results will add to the value of peptidomic analysis as a potential technology to discover serum biomarkers of cartilage degradation and will aid in further understanding of the underlying mechanisms of OA.

Takinami et al. [28] conducted a study on a total of 69 plasma samples (25 OA patients with radiographic progression, 33 nonprogressive OA patients, and 11 healthy donors). Three biomarkers significantly differentiate between progressor and nonprogressor OA. Moreover, they used MALDI-TOF-MS combined with surface-enhanced laser desorption/ionization time-of-flight mass spectrometry (SELDI-TOF-MS), and subsequent analyses indicated that these peaks corresponded to apolipoprotein C-I and C-III and an N-terminal truncated form of transthyretin, respectively. They reported that these 3 peaks are expected to be prognostic biomarkers for knee OA, capable of predicting the progression of knee OA and facilitating the development of novel disease-modifying treatments for OA.

In another study [29], sera were used from moderate and severe OA patients and compared to healthy controls. Serum protein levels were analyzed using isobaric tags for relative and absolute quantitation (iTRAQ) and MALDI-TOF-MS. More than 300 different proteins were isolated from sera of OA patients, and more than 250 have been quantified by calculation of their iTRAQ ratios. Three sets of proteins were significantly changed in OA samples compared to controls. These included some complement components, lipoproteins, von Willebrand factor, tetranectin, and lumican.

In our study, the Receiver Operating Characteristic (ROC) curve has been used to test the accuracy of the training set data in diagnosing RA. Comparing the detected protein peaks of RA patients (35 samples) with those of overall control subjects (70 samples) showed that the significant integrated peaks could differentiate RA from control subjects (OA and HC) with sensitivity and specificity of 96.66% and 100.0%, respectively, with an AUC = 0.988. That was higher than the diagnostic performance of the anti-citrullinated peptide antibody (ACPA) assay with a sensitivity of 83.43% and specificity of 86.67% (AUC = 0.875) and rheumatoid factor (RF) assay with a sensitivity of 80.9% and specificity of 46.67% (AUC = 0.720) in diagnosing RA. Thus, the good performance indicated that the detected protein peaks could be potential diagnostic biomarkers for RA and could effectively distinguish RA patients from individuals with osteoarthritis or healthy people.

Although MALDI-TOF-MS looks very promising, it is still in its infancy. Because proteomics expresses the overall picture of the intracellular protein structure, it is capable of identifying noninvasive diagnostic biomarkers, giving the chance for further molecular researches, and improving the understanding of RA pathogenesis. However, utilization of MALDI-TOF-MS analysis requires skilled personnel and subsequently increases the costs; therefore, their broad use especially in the developing countries is limited.

## 5. Conclusion

MALDI-TOF-MS combined with MB-HIC C8 is a potentially effective technology to identify novel serum protein biomarkers related to RA. The present data suggested that the peptidomic pattern is of value for differentiating individuals with RA from OA and healthy controls.

## Figures and Tables

**Table 1:** Characteristics of participants.

	RA group (*n* = 35)	OA group (*n* = 35)	HC group (*n* = 35)	Test of sig.	*p*
Gender					
Female (male)	24 (11)	26 (9)	26 (9)	*χ* ^2^ = 3.205	^MC^ *p* = 0.194
Age					
Min.–Max.	27–58	40–62	28–60	*H* = 0.11	0.95
Mean ± SD	32.7 ± 10.3	45.24 ± 9.15	35 ± 8.2		
ESR (1^st^ hour)	46.5 ± 16.4	22.9 ± 5.5	19.4 ± 4.8	*H* = 69.5^∗^	<0.001^∗^
Sig.bet.Gps	*p*_1_ < 0.001^∗^, *p*_2_ < 0.001^∗^, *p*_3_ < 0.001^∗^				
CRP (<6 IU/ml)	19.1 ± 5.9	6.2 ± 1.8	3.1 ± 1.6	*H* = 57.4^∗^	<0.001^∗^
Sig.bet.Gps	*p*_1_ < 0.001^∗^, *p*_2_ < 0.001^∗^, *p*_3_ < 0.001^∗^				
ALT (18-37 *μ*/l)	27.3 ± 10.4	23.2 ± 3.4	25.1 ± 5.3	*H* = 1.68	0.48
AST (22-38 *μ*/l)	30.6 ± 9.9	29.5 ± 5.2	27.7 ± 4.3	*H* = 2.16	0.35
S. urea (15-45 mg/dl)	28.7 ± 8.76	31.3 ± 4.5	20.6 ± 6.7	*H* = 1.74	0.27
S. creatinine (0.5–1.2 mg/dl)	0.7 ± 0.2	0.9 ± 0.4	0.8 ± 0.3	*H* = 0.18	0.93
Hb (12-17 g/dl)	10.1 ± 1.5	11.4 ± 1.2	13.3 ± 0.7	*H* = 4.94	0.08
WBCs (4‐11 × 10^3^/mm^3^)	6.5 ± 1.9	7.4 ± 1.9	7.8 ± 2.4	*H* = 1.55	0.56
Platelets (150‐450 × 10^3^/mm^3^)	347.4 ± 86.4	277.4 ± 70.4	248.8 ± 29.5	*H* = 5.83	0.07

RA: rheumatoid arthritis; OA: osteoarthritis; HC: healthy control; ESR: erythrocyte sedimentation rate; CRP: C-reactive protein; ALT: alanine transaminase; AST: aspartate transaminase; Hb: hemoglobin; WBC: white blood cells. *H*: *H* for the Kruskal-Wallis test; pairwise comparison between every 2 groups was done using a post hoc test (Dunn's for multiple comparison test). ^∗^Statistically significant at *p* ≤ 0.05. *χ*^2^: Chi-squared test; MC: Monte Carlo; *H*: *H* for Kruskal-Wallis test; *U*: Mann–Whitney test. *p*_1_: *p* value for comparison between RA and OA groups. *p*_2_: *p* value for comparison between RA and HC groups. *p*_3_: *p* value for comparison between OA and HC groups.

**Table 2:** Disease parameters of the rheumatoid arthritis group.

	RA group (*n* = 35) Mean ± SD
Disease duration (years)	4.7 ± 2.6
DAS-28	4.1 ± 0.9
RF (*μ*/ml)	112 ± 34
ACPA (*μ*/ml)	248 ± 59

DAS-28: disease activity score 28; RF: rheumatoid factor; ACPA: anti-cyclic citrullinated peptide antibody.

**Table 3:** Serum peptidomic profile of the three training sets.

Total number of peaks	No. of masses of integrating regions	No. of significant peaks	No. of significant integrated peaks	Sensitivity	Specificity
RA versus OA groups					
113	75 : 7767.82	5	5	97.8%	97.9%
	40 : 2953.29				
	41 : 2991.59				
	48 : 4054.75				
	68 : 6434.51				

RA versus HC groups					
101	25 : 2367.5	53	3	97.5%	95.3%
	62 : 7767.8				
	53 : 5906.33				
	14 : 1617.46				
	43 : 4210.99				

OA versus HC groups					
95	18 : 2953.08	29	3	96.6%	99.7%
	03 : 1077.37				
	75 : 9289.67				
	14 : 2272.81				
	23 : 3316.27				

RA: rheumatoid arthritis; OA: osteoarthritis; HC: healthy control; *p* < 0.05.

**Table 4:** Comparison of training set data with other laboratory markers for diagnosing RA.

	RA serum protein peaks	ACPA	RF
Sensitivity (95% CI)	96.66	83.43	80.9
Specificity (95% CI)	100.0	86.67	46.67
PPV	100.0	86.2	61.3
NPV	96.8	83.9	72.0
*p*	<0.001^∗^	<0.001^∗^	0.004^∗^
AUC (95% CI)	0.988	0.875	0.720

RA: rheumatoid arthritis; RF: rheumatoid factor; ACPA: anti-cyclic citrullinated peptide antibody; CI: confidence intervals; NPV: negative predictive value; PPV: positive predictive value; AUC: area under the curve. ∗: statistically significant at *p* ≤ 0.05.

## Data Availability

The data used to support the findings of this study are available from the first author/corresponding author upon request.

## References

[1] Smolen J. S., Aletaha D. (2004). Patients with rheumatoid arthritis in clinical care. *Annals of Rheumatic Diseases*.

[2] Abdelati A., Elnemr R., Ismail A., Gamal-Eldeen M. (2019). TCR-CD3*ζ* gene expression profile in patients with rheumatoid arthritis and correlation with disease activity. *Egyptian Rheumatology & Rehabilitation*.

[3] Landewé R. B. M. (2003). The benefits of early treatment in rheumatoid arthritis: confounding by indication and the issue of timing. *Arthritis and Rheumatism*.

[4] Aletaha D., Neogi T., Silman A. J. (2010). 2010 Rheumatoid Arthritis Classification criteria: An American College of Rheumatology/European League Against Rheumatism collaborative initiative. *Arthritis and Rheumatism*.

[5] Park Y. J., Chung M. K., Hwang D., Kim W. U. (2015). Proteomics in rheumatoid arthritis research. *Immune Network*.

[6] Cheng Y., Chen Y., Sun X. (2014). Identification of potential serum biomarkers for rheumatoid arthritis by high-resolution quantitative proteomic analysis. *Inflammation*.

[7] Vanarsa K., Mohan C. (2010). Proteomics in rheumatology: the dawn of a new era. *F1000 Medicine Reports*.

[8] Yan Z., Chaojun H., Chuiwen D. (2015). Establishing serological classification tree model in rheumatoid arthritis using combination of MALDI-TOF-MS and magnetic beads. *Clinical and Experimental Medicine*.

[9] Long L., Li R., Li Y., Hu C., Li Z. (2011). Pattern-based diagnosis and screening of differentially expressed serum proteins for rheumatoid arthritis by proteomic fingerprinting. *Rheumatology International*.

[10] Castro-Santos P., Laborde C. M., Dıaz-Pena R. (2015). Genomics, proteomics and metabolomics: their emerging roles in the discovery and validation of rheumatoid arthritis biomarkers. *Clinical and Experimental Rheumatology*.

[11] Kang M. J., Park Y. J., You S. (2014). Urinary proteome profile predictive of disease activity in rheumatoid arthritis. *Journal of Proteome Research*.

[12] Liao H., Wu J., Kuhn E. (2004). Use of mass spectrometry to identify protein biomarkers of disease severity in the synovial fluid and serum of patients with rheumatoid arthritis. *Arthritis and Rheumatism*.

[13] Kellgren J. H., Lawrence J. S. (1957). Radiological assessment of osteoarthrosis. *Annals of Rheumatic Diseases*.

[14] Prevoo M. L. L., van'T Hof M. A., Kuper H. H., van Leeuwen M. A., van de Putte L. B. A., van Riel P. L. C. M. (1995). Modified disease activity scores that include twenty-eight-joint counts Development and validation in a prospective longitudinal study of patients with rheumatoid arthritis. *Arthritis and Rheumatism*.

[15] Lorenz P., Ruschpler P., Koczan D., Stiehl P., Thiesen H. J. (2003). From transcriptome to proteome: differentially expressed proteins identified in synovial tissue of patients suffering from rheumatoid arthritis and osteoarthritis by an initial screen with a panel of 791 antibodies. *Proteomics*.

[16] Kandil N. S., Ghazala R. A., El Sharkawy R. M., Youssif T. A., Abouseda N. N. (2019). Evaluation of protein profiling in a cohort of Egyptian population with renal cell carcinoma and benign kidney neoplasms. *Asian Pacific Journal of Cancer Prevention*.

[17] Smolen J. S., Aletaha D., McInnes I. B. (2016). Rheumatoid arthritis. *The Lancet*.

[18] Baillet A., Trocme C., Berthier S. (2010). Synovial fluid proteomic fingerprint: S100A8, S100A9 and S100A12 proteins discriminate rheumatoid arthritis from other inflammatory joint diseases. *Rheumatology*.

[19] Maecker H. T., Lindstrom T. M., Robinson W. H. (2012). New tools for classification and monitoring of autoimmune diseases. *Nature Reviews Rheumatology*.

[20] Xinqiang S., Kaiming L., Lei C., Lim T. K., Lee Y. M., Yuan L. (2018). Quantitative proteomic analysis of peripheral blood mononuclear cells in rheumatoid arthritis. *Rheumatology and Orthopedic Medicine*.

[21] Whiteaker J. R., Zhao L., Zhang H. Y. (2007). Antibody-based enrichment of peptides on magnetic beads for mass-spectrometry- based quantification of serum biomarkers. *Analytical Biochemistry*.

[22] Sinz A., Bantscheff M., Mikkat S. (2002). mass spectrometric proteome analyses of synovial fluids and plasmas from patients suffering from rheumatoid arthritis and comparison to reactive arthritis or osteoarthritis. *Electrophoresis*.

[23] Tsuno H., Arito M., Suematsu N. (2018). A Proteomic analysis of serum-derived exosomes in rheumatoid arthritis. *BMC Rheumatology*.

[24] Niu Q., Huang Z., Shi Y., Wang L., Pan X., Hu C. (2010). Specific serum protein biomarkers of rheumatoid arthritis detected by MALDI-TOF-MS combined with magnetic beads. *International Immunology*.

[25] Zhang X., Yuan Z., Shen B., Zhu M., Liu C., Xu W. (2012). Discovery of serum protein biomarkers in rheumatoid arthritis using MALDI-TOF-MS combined with magnetic beads. *Clinical and Experimental Medicine*.

[26] Li Y., Sun X., Zhang X. (2015). Establishment of a decision tree model for diagnosis of early rheumatoid arthritis by proteomic fingerprinting. *International Journal of Rheumatic Diseases*.

[27] Kabeerdoss J., Kurien B. T., Ganapati A., Danda D. (2015). Proteomics in rheumatology. *International Journal of Rheumatic Diseases*.

[28] Takinami Y., Yoshimatsu S., Uchiumi T. (2013). Identification of potential prognostic markers for knee osteoarthritis by serum proteomic analysis. *Biomark Insights*.

[29] Fernández-Puente P., Mateos J., Fernández-Costa C. (2011). Identification of a panel of novel serum osteoarthritis biomarkers. *Journal of Proteome Research*.

